# Quantitative biomechanical analysis of fracture patterns in ipsilateral femoral neck and shaft fractures: an in-silico study

**DOI:** 10.3389/fbioe.2025.1641700

**Published:** 2025-08-22

**Authors:** Zhongwei Xiong, Zihao Liu, Houquan Zhou, Xiang Zhang, Jialei Chen

**Affiliations:** ^1^ Department of Orthopedics, Luzhou Longmatan District People’s Hospital, Luzhou, Sichuan, China; ^2^ Orthopaedic Trauma, Harrison International Peace Hospital, Hengshui, Hebei, China; ^3^ Department of Orthopaedics, West China Hospital, Sichuan University, Chengdu, Sichuan, China

**Keywords:** ipsilateral femoral neck and shaft fractures, biomechanics, finite element analysis, evaluation parameters, fixation stability

## Abstract

**Introduction:**

Ipsilateral femoral neck and shaft fractures (IFN-SFs) caused by high-energy trauma pose a significant risk of complications related to bone healing. Prompt identification of fracture types and maintenance of fracture fixation stability can mitigate this risk. This study employed finite element analysis to evaluate biomechanical parameters for the stability of fixation in IFN-SFs and quantify differences in biomechanical stability among various fracture types.

**Methods:**

Patient-specific femur models were constructed from computed tomography data of 10 individuals. Simulating combinations of three Pauwels classifications (I-III) of femoral neck fractures (FNFs) and three femoral shaft fracture (FSF) types (transverse, wedge-shaped, comminuted), a total of 90 FNF-FSF models were generated. Reconstruction nails with cannulated screws were used to fix the fracture models, and postoperative single-leg standing loads were simulated. The entropy method comprehensively evaluated biomechanical parameters to assess the fixation stability for different fracture types.

**Results:**

Among fracture types, Pauwels Type III FNF combined with comminuted FSF showed the lowest stability (composite score = 0.22), while Pauwels Type I FNF with transverse FSF was the most stable (composite score = 0.79). Maximum implant stress (weight = 24.5%) and maximum proximal-distal femur interfragmentary motion (weight = 17.1%) were the most influential biomechanical parameters for assessing IFN-SF stability.

**Conclusion:**

For IFN-SFs, the FNF type primarily dictates fixation stability, with the FSF contributing synergistically. Maximum implant stress and proximal-distal femur interfragmentary motion are key biomechanical indicators for assessing IFN-SFs. Personalized enhanced fixation strategies are essential for ipsilateral Pauwels Type III FNF and comminuted FSF.

## 1 Introduction

Ipsilateral femoral neck and shaft fractures (IFN-SFs) are uncommon, accounting for less than 10% of all femoral shaft fractures (FSFs) (Boulton and Pollak, 2015). These fractures primarily result from axial loads on the femoral shaft during high-speed motor vehicle accidents. Femoral neck fractures (FNFs) are frequently overlooked during diagnosis and treatment, increasing the risk of post-operative non-union and femoral head necrosis to 10% and 22%, respectively (Mohan et al., 2019). Additionally, the deterioration of the biomechanical environment at the fracture site is a key factor contributing to non-union of FSFs (Mimata et al., 2022). Complications related to fracture fixation are closely associated with the complex mechanical characteristics of the fracture site. Therefore, understanding the biomechanical mechanism of fracture fixation is crucial for reducing the risk of complications.

The treatment options for IFN-SFs remain controversial (Bastian et al., 2023). Traditional clinical studies have primarily focused on exploring the optimal fixation methods for these fractures, categorizing fixation strategies into single and dual fixation types (Hak et al., 2015). Studies comparing single versus dual fixation in IFN-SF patients have not revealed significant discrepancies in postoperative fracture healing time, healing rates, healing-related complications, or functional outcomes (Mohan et al., 2019; Zhao et al., 2023). This suggests that the differences in biomechanical stability among different fracture types may be the key factor influencing the risk of postoperative complications in patients. Previous studies have attempted to evaluate treatment outcomes through fracture classification, but due to the low incidence of IFN-SFs and the limited types of fractures included, the conclusions drawn are not fully generalizable (Gupta et al., 2023). Most simulation and biomechanical studies have focused on the influence and differences of different fixation methods on the stability of IFN-SFs, but there is insufficient research on the biomechanical evidence of fixation stability for different fracture types (Yuan et al., 2022; Xiao et al., 2025). Therefore, further investigation into the biomechanical outcomes of fixation structures for different fracture types of IFN-SFs is particularly necessary.

Given the multitude of biomechanical parameters used to assess femoral neck and shaft fractures, a direct comparison of each indicator may not be entirely convincing. Xiao et al. (2025) analyzed stress distribution in the femoral neck, fracture surfaces of the femoral shaft and implants, and the displacement of bones and implants to determine the optimal fixation strategy for IFN-SFs. Other studies have included parameters such as maximum stress and displacement of bones and implants, stiffness of the bone-implant structure, interfragmentary motion, compressive stress, and interfragmentary gap in their evaluations of fracture stability (Jung et al., 2022; Zhong et al., 2023). Comprehensive consideration of each parameter is crucial for accurately identifying the biomechanical indicators for evaluating fixation stability in IFN-SFs. Therefore, this study develops finite element models of IFN-SFs with different fracture types to determine appropriate biomechanical parameters for evaluating fixation stability and to investigate differences in biomechanical stability of fixation structures for various fracture types.

## 2 Materials and methods

### 2.1 Participants

The study protocol was reviewed and approved by the Biomedical Research Ethics Committee of our hospital (IRB #2024–724). Before initiating the research, we used Gpower software (Heinrich Heine Universitat Düsseldorf, Germany) to calculate and determined that the minimum sample size required for this study protocol was 8 (α = 0.05, β = 0.2, f = 0.42). Considering the reproducibility of the FEA model and the representativeness of the clinical data, the study included computed tomography data from the healthy femurs of 10 patients diagnosed with IFN-SF at our institution (Supplementary Table S1). None of the patients had osteoarthritis, bone tumors, or other metabolic diseases at the time of CT scanning. Given the retrospective nature of the study, the Ethics Committee waived the requirement for written informed consent.

### 2.2 FEA modeling

**FIGURE 1:**
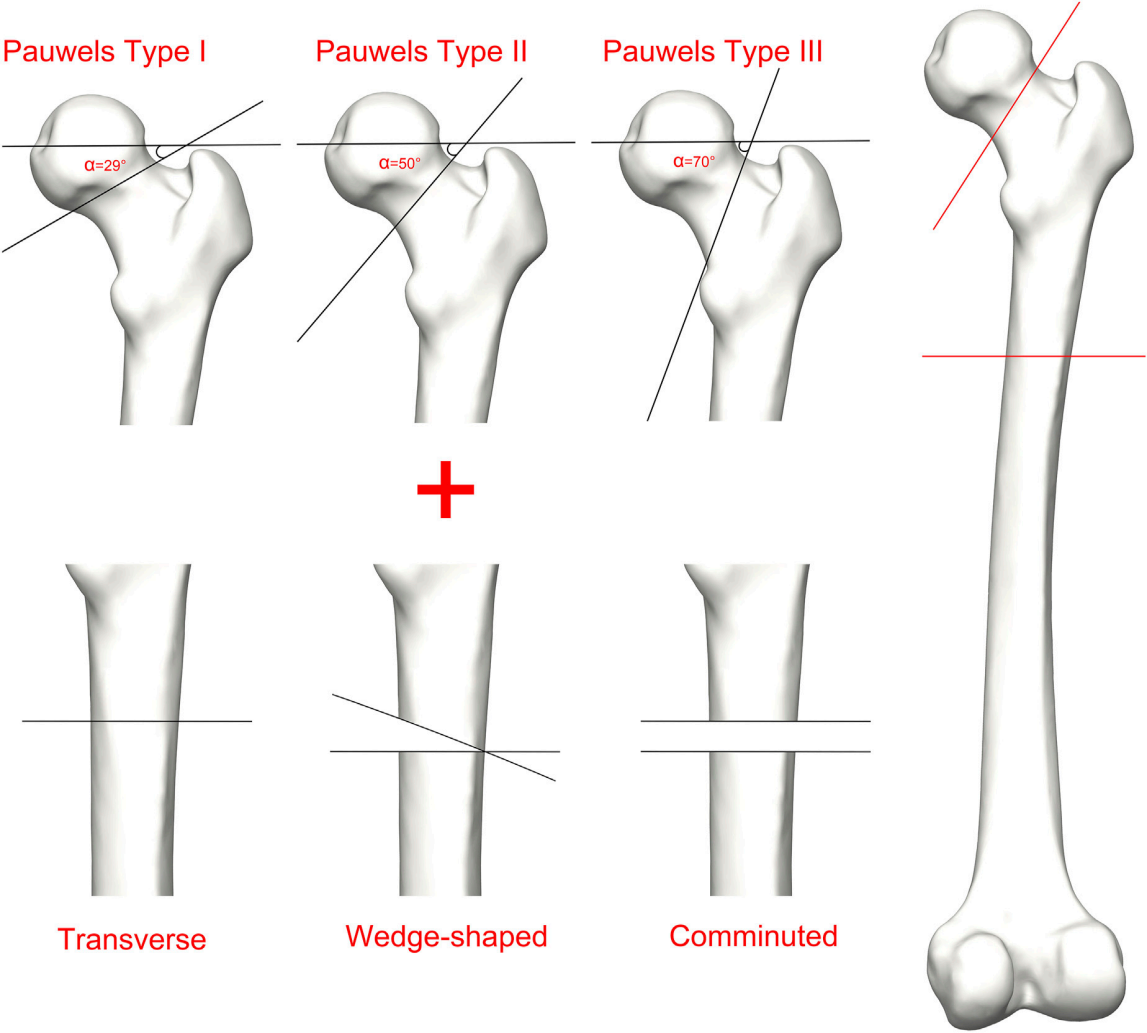
Schematic of Ipsilateral femoral neck and shaft fractures. Fractures of the femoral neck are classified as Pauwels I, Pauwels II, and Pauwels III; Fractures of the femoral shaft are classified as transverse, wedge-shaped, and comminuted.

In accordance with the research protocol described by Peng et al. (2020), the Mimics software (Materialise Group, Leuven, Belgium) was employed to process the CT data of the healthy-side femurs of ten patients participating in the study, generating patient-specific three-dimensional models of both cortical and cancellous bone. Subsequently, the femur models were imported into Geomagic software (Geomagic, United States) for additional refinement. Then, the models were imported into SolidWorks 2021 (Dassault, France) to create models of IFN-SFs. As indicated by Marins et al. (2021), in instances of IFN-SFs, the FNFs predominantly exhibit type B2 characteristics, and the most prevalent types of FSFs are classified as A3, B, and C. Most biomechanical studies utilize the Pauwels classification to quantify the biomechanical loading characteristics on FNFs (Zeng et al., 2020; Xia et al., 2021). In this study, we employed SolidWorks software to generate ten patient-specific femur models representing Pauwels type I, II, and III FNFs. Furthermore, to investigate the impact of FSF type, we developed transverse, wedge, and comminuted FSF models (Figure 1). These fracture models were then grouped into nine categories, resulting in a total of 90 fracture models: Pauwels type I FNF and transverse FSF (I-T), Pauwels type I FNF and wedge-shaped FSF (I-W), Pauwels type I FNF and comminuted FSF (I-C), Pauwels type II FNF and transverse FSF (II-T), Pauwels type II FNF and wedge-shaped FSF (II-W), Pauwels type II FNF and comminuted FSF (II-C), Pauwels type III FNF and transverse FSF (III-T), Pauwels type III FNF and wedge-shaped FSF (III-W), and Pauwels type III FNF and comminuted FSF (III-C).

The biomechanical study conducted by Wu and Tai (2009) suggested that incorporating a cannulated screw into reconstruction nail fixation could improve the stability of IFN-SF fixation. Therefore, based on the dimensions provided by manufacture, we used Solidworks software to developed three-dimensional solid models of the reconstruction nail and cannulated screw. The reconstruction nail had a diameter of 12 mm, with its length customized based on the femur length of patients. The cannulated screw design featured 7 mm diameter partially threaded screws, with simplified threads to mitigate stress concentration errors. Subsequently, fixation and assembly procedures were carried out following established surgical protocols. The cannulated screw model was placed in the anterior third of the femoral neck, while the reconstruction nail was positioned in the piriform fossa at the posterior third of the proximal femur (Spitler et al., 2020). The length of the implant and the fixation angle were customized for each femur model in the sample.

### 2.3 FE parameter setting

All models were assumed to be linearly elastic materials. Using the Mimics software, the elastic moduli of cortical and cancellous bone for each femur sample were determined by applying a formula that relies on the CT Hounsfield Unit (HU) values of the femurs from the ten patients (Jian-Qiao Peng et al., 2020):

$$E_{B}\;\left(\text{MPa}\right)=0.004\;{\left(131–1.067\text{HU}\right)}^{2.01}$$

EB is the elastic modulus of bone, and the Poisson’s ratio (ν) of bone was set to 0.3. All screw and reconstruction nail models were defined as titanium alloy (Ti - 6AL - 7Nb) materials, with a Young’s modulus of 110 GPa and a Poisson’s ratio of 0.3 (Huang et al., 2021).

The models were meshed using quadratic tetrahedral elements (C3D10) in Ansys Workbench 202R2. Mesh convergence analysis established the optimal mesh size for calculations (Huang et al., 2023). Increasing the number of mesh elements and nodes had a negligible effect on the Von Mises stress (VMS) values of the implant and femur, staying within a 5% variation (Supplementary Table S2; Supplementary Figure S1). Therefore, mesh sizes of 2 mm were designated for model meshing. The quantity of mesh elements (856,333 to 863,502) and nodes (561,888 to 566,405) varied among different solid models.

**FIGURE 2:**
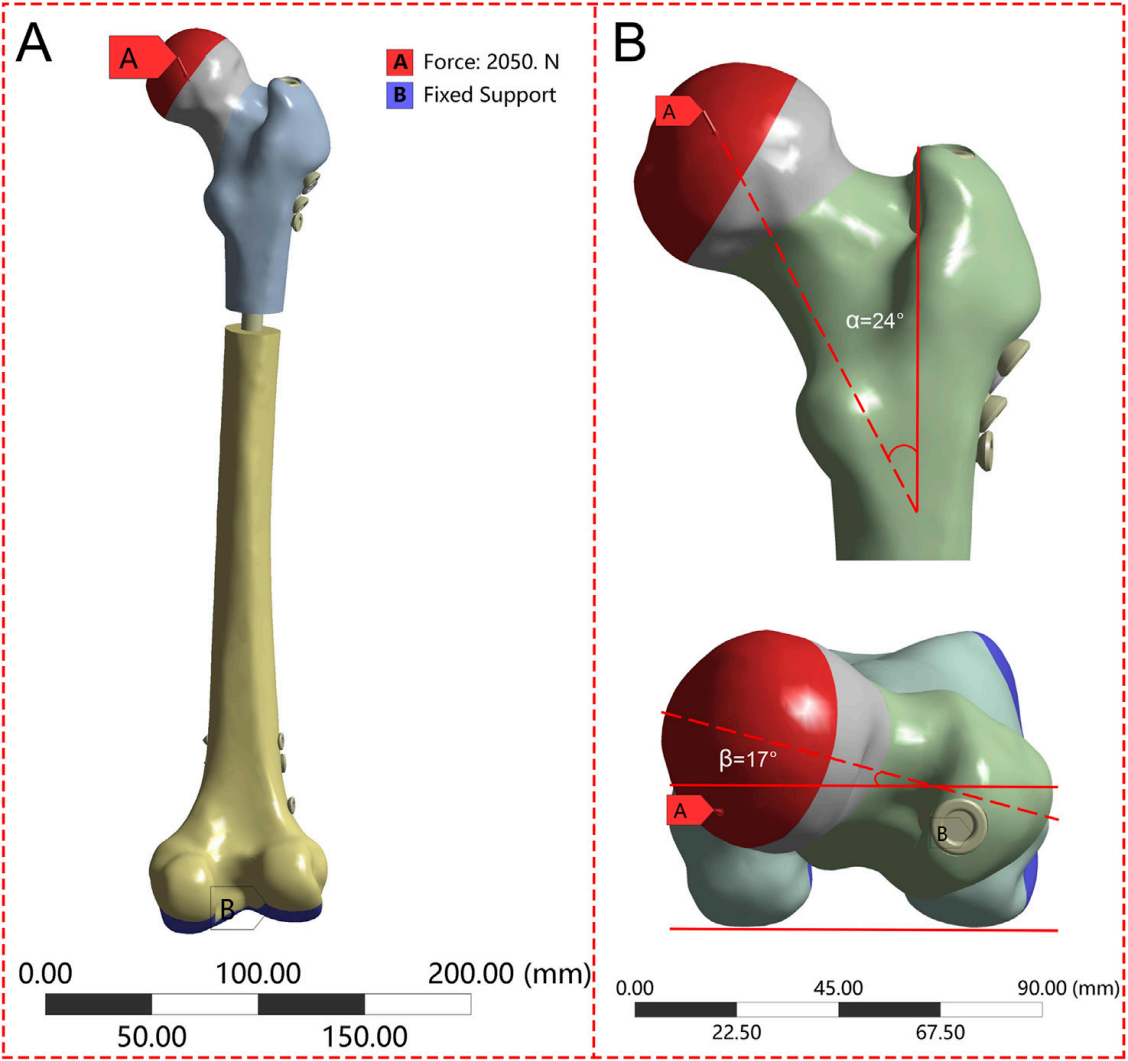
The femur underwent loading during the single-leg stance in each finite element model. **(A)** Schematic diagram of the overall boundary conditions; **(B)** The load vector was oriented at 24° in the frontal plane and 17° in the axial plane.

The boundary conditions were set based on the methodology employed in prior research (Figure 2). The interface between the implant and bone was specified as a bonded contact, whereas the contact between the fracture surfaces was defined as a frictional contact with a friction coefficient of 0.3 (Yuan et al., 2022). To replicate the peak load encountered by the hip joint during walking, the degrees of freedom of the distal femur were restricted, and a load equal to 300% of the individual’s body weight was exerted on the femoral head along the mechanical axis of the femur (Jung et al., 2022).

### 2.4 Assessment of biomechanical parameters

**FIGURE 3:**
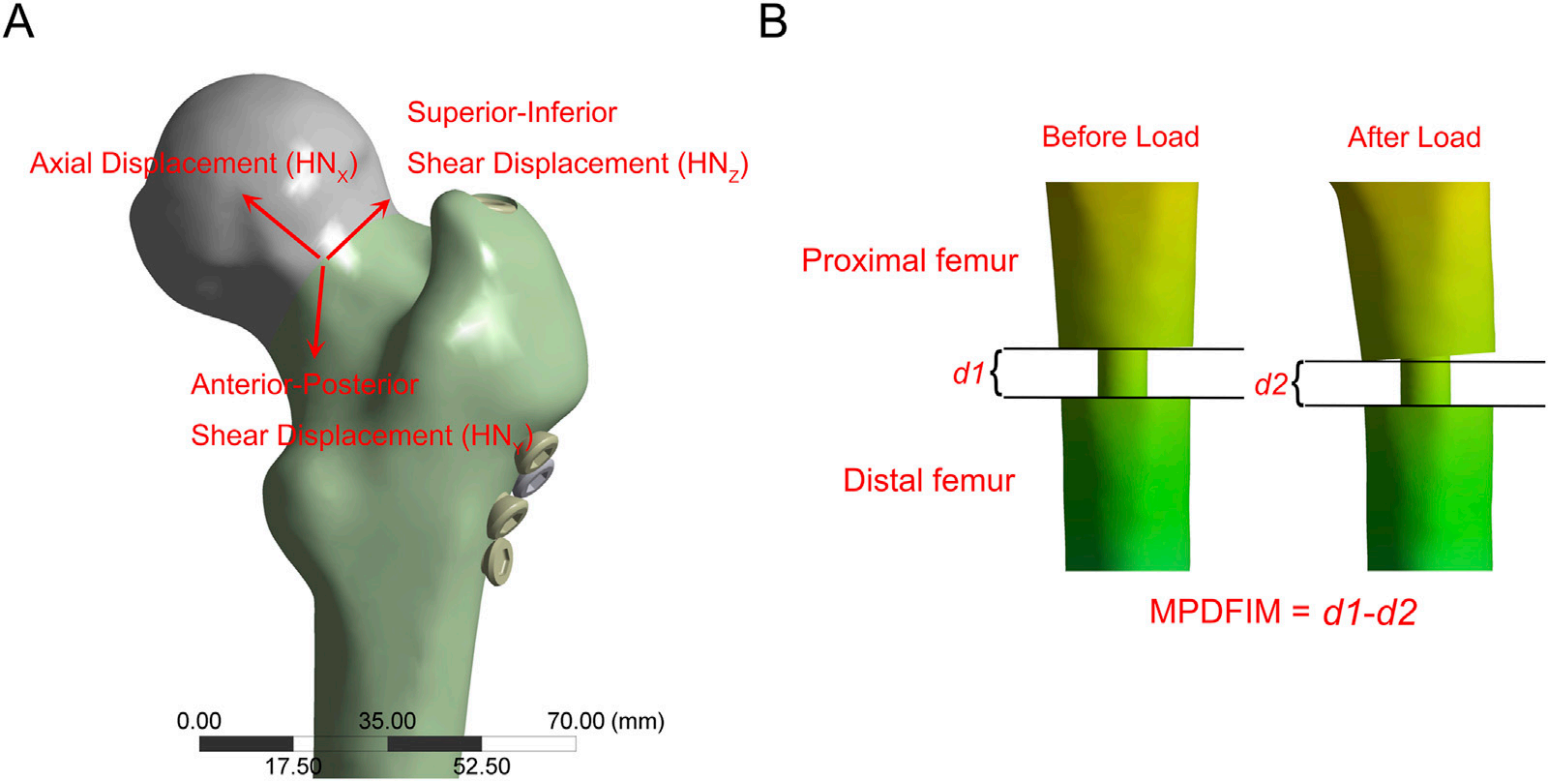
The schematic diagram illustrates the relative displacement. **(A)** The maximum femoral head-neck interfragmentary motion (MFHNIM) is determined by assessing the relative displacement between the femoral head and femoral neck fracture fragments along the X, Y, and Z-axes; **(B)** The maximum proximal-distal femur interfragmentary motion (MPDFIM) is determined by evaluating the variation in fracture gaps before and after load application.

Based on prior research, we evaluated nine biomechanical parameters related to fracture fixation stability (Samsami et al., 2019; Peng et al., 2020; Xia et al., 2021; Jung et al., 2022). These parameters comprised stiffness (load divided by displacement), the maximum implant von Mises stress (MIVMS), the maximum implant displacement (MID), the maximum proximal femur von Mises stress (MPFVMS), the maximum distal femur von Mises stress (MDFVMS), the maximum femur displacement (MFD), the maximum femoral neck von Mises stress (MFNVMS), the maximum femoral head-neck interfragmentary motion (MFHNIM), and the maximum proximal-distal femur interfragmentary motion (MPDFIM). The femoral head-neck interfragmentary motion (Figure 3A) was calculated using following Equation (Samsami et al., 2019):

$$MFHNIM=\sqrt{{\text{HN}}_{X}^{2}+{\text{HN}}_{Y}^{2}+{\text{HN}}_{Z}^{2}}$$

HN_X_, HN_Y_, and HN_Z_ denote the relative displacements of the femoral head in the X, Y, and Z directions, respectively, concerning the femoral neck. The PDFIM represents the relative displacement between the proximal and distal femurs along the vertical axis (Figure 3B).

### 2.5 Statistical analysis

Statistical analyses were conducted using SPSS 22 (IBM Corp., Armonk, NY, United States) and GraphPad Prism 10.4 (GraphPad Software, San Diego, CA, United States). FEA results were reported as mean ± standard deviation. Normality and homogeneity of variance were assessed using Shapiro-Wilk and Levene’s tests, respectively. A two-way repeated measures ANOVA was employed to assess the impact of the interaction between FNF and FSF types on biomechanical parameters. Subsequently, the entropy method was used to rank the results of various biomechanical parameters. A p-value <0.05 was considered statistically significant.

## 3 Results

### 3.1 Effects of FNFs and FSFs on biomechanical parameters

Table 1 demonstrates that the type of FNF had a significant impact on the outcomes of MIVMS, MID, MPFVMS, MFNVMS, and MFHNIM (p < 0.01). FSF significantly influenced the results of MIVMS, MPFVMS, MDFVMS, and MPDFIM (p < 0.01). The interaction between the two fracture types primarily affected MIVMS, MPFVMS, and MDFVMS (p < 0.01).

**TABLE 1:** Results of two-way repeated measures analysis of variance for nine biomechanical parameters.

	FNF type		FSF type		FNF*FSF	
	F	p	F	p	F	p
Stiffness (N/mm)	0.13	0.88	0.29	0.75	0.02	1.00
MIVMS (Mpa)	10.74	0.00*	911.17	0.00*	9.12	0.00*
MID (mm)	0.19	0.83	0.38	0.69	0.05	1.00
MPFVMS (MPa)	34.21	0.00*	431.88	0.00*	20.97	0.00*
MDFVMS (MPa)	0.29	0.75	156.01	0.00*	8.73	0.00*
MFD (mm)	0.19	0.82	0.37	0.69	0.05	1.00
MFNVMS (MPa)	180.52	0.00*	0.09	0.91	0.42	0.80
MFHNIM (mm)	208.85	0.00*	1.11	0.34	0.35	0.84
MPDFIM (mm)	0.03	0.97	1374.23	0.00*	0.04	1.00

*p < 0.05.

### 3.2 Biomechanical parameters

**FIGURE 4:**
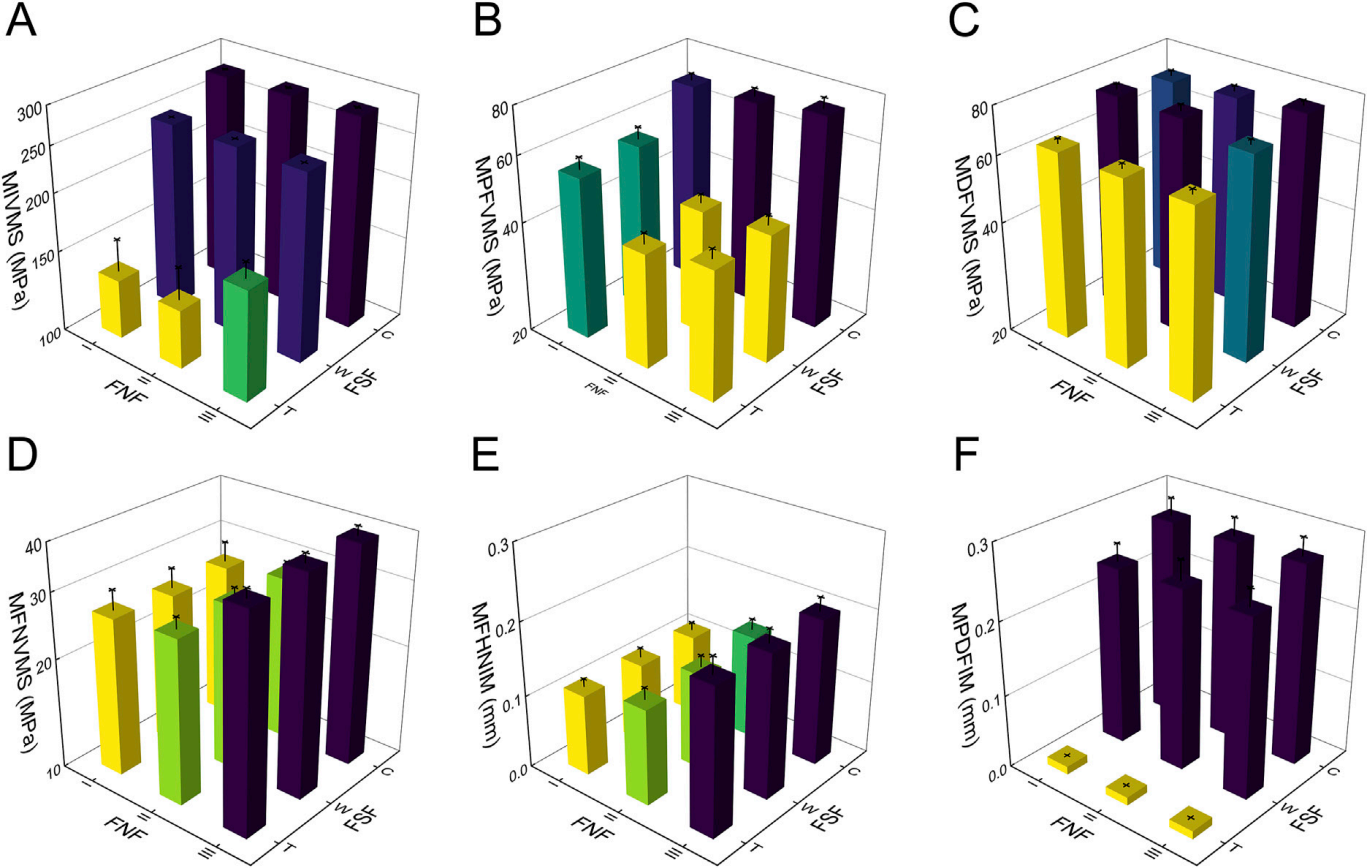
Results of finite element analysis for different IFN-SF types under single-leg standing load. **(A)** (MIVMS) the maximum implant von Mises stress, **(B)** (MPFVMS) the maximum proximal femur von Mises stress, **(C)** (MDFVMS) the maximum distal femur von Mises stress, **(D)** (MFNVMS) the maximum femoral neck von Mises stress, **(E)** (MFHNIM) the maximum femoral head-neck interfragmentary motion, **(F)** (MPDFIM) and the maximum proximal-distal femur interfragmentary motion.

**FIGURE 5:**
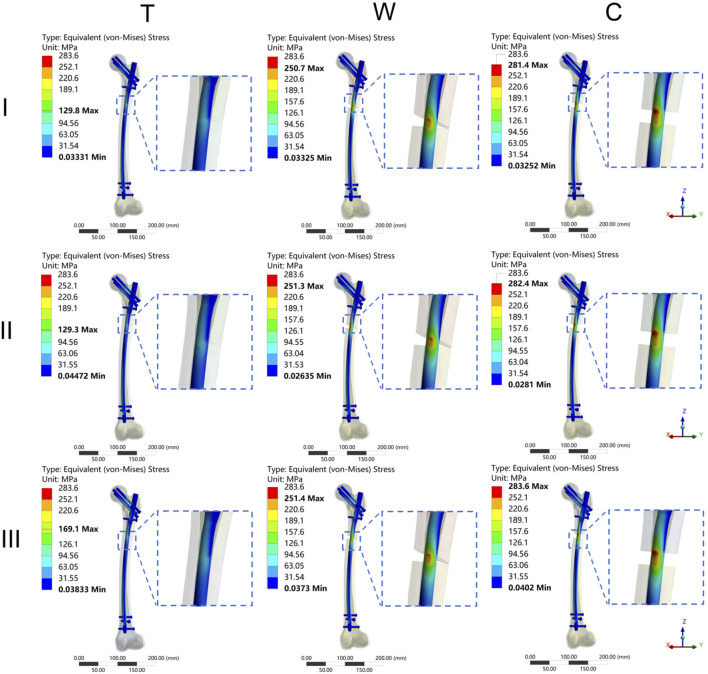
The cloud diagram of the maximum implant von Mises stress. I: Pauwels type I femoral neck fracture; II: Pauwels type II femoral neck fracture; III: Pauwels type III femoral neck fracture; T: Transverse femoral shaft fracture, W: wedge-shaped femoral shaft fracture, C: comminuted femoral shaft fracture.

**FIGURE 6:**
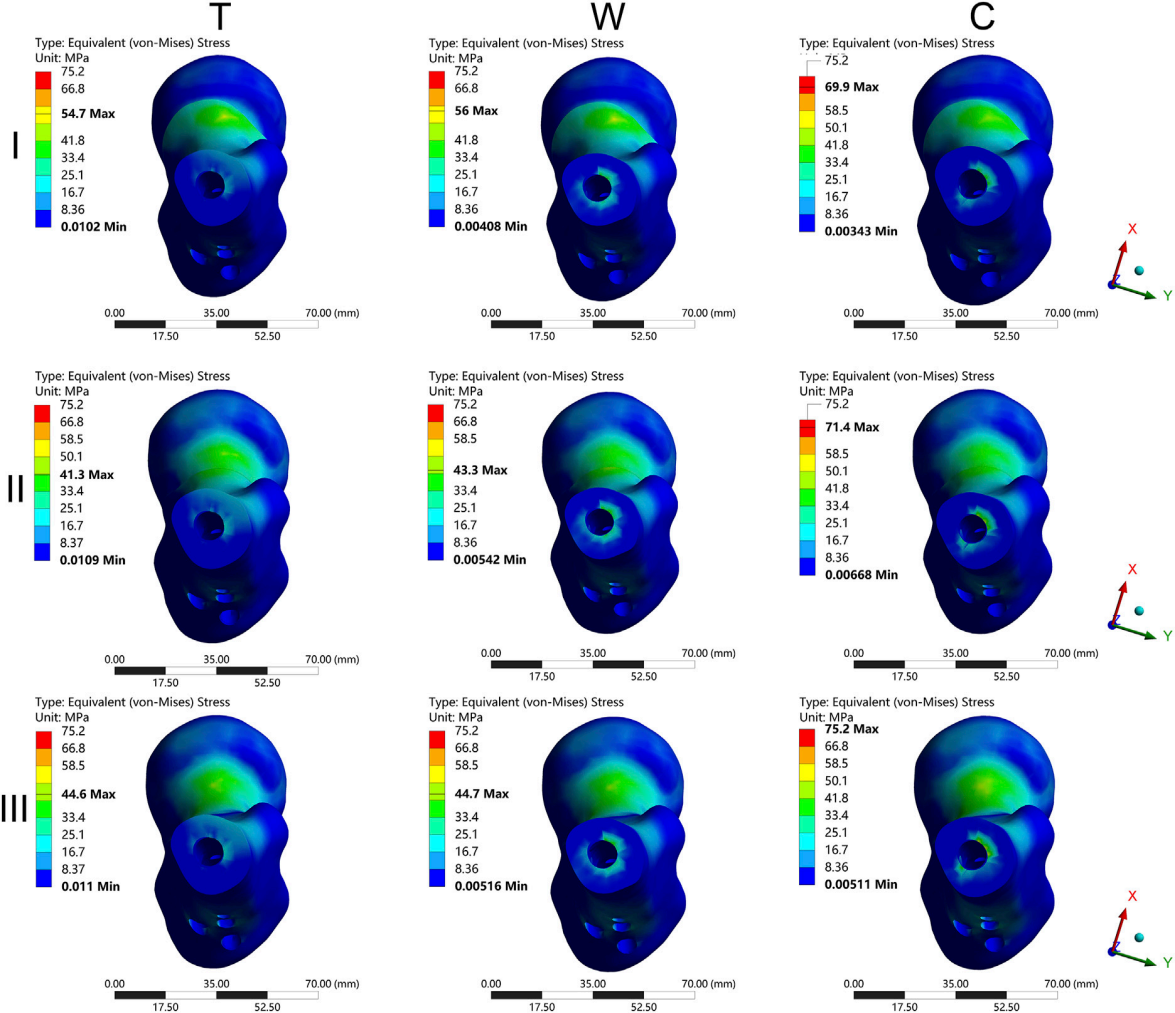
The cloud diagram of the maximum proximal femur von Mises stress. I: Pauwels type I femoral neck fracture; II: Pauwels type II femoral neck fracture; III: Pauwels type III femoral neck fracture; T: Transverse femoral shaft fracture, W: wedge-shaped femoral shaft fracture, C: comminuted femoral shaft fracture.

**FIGURE 7:**
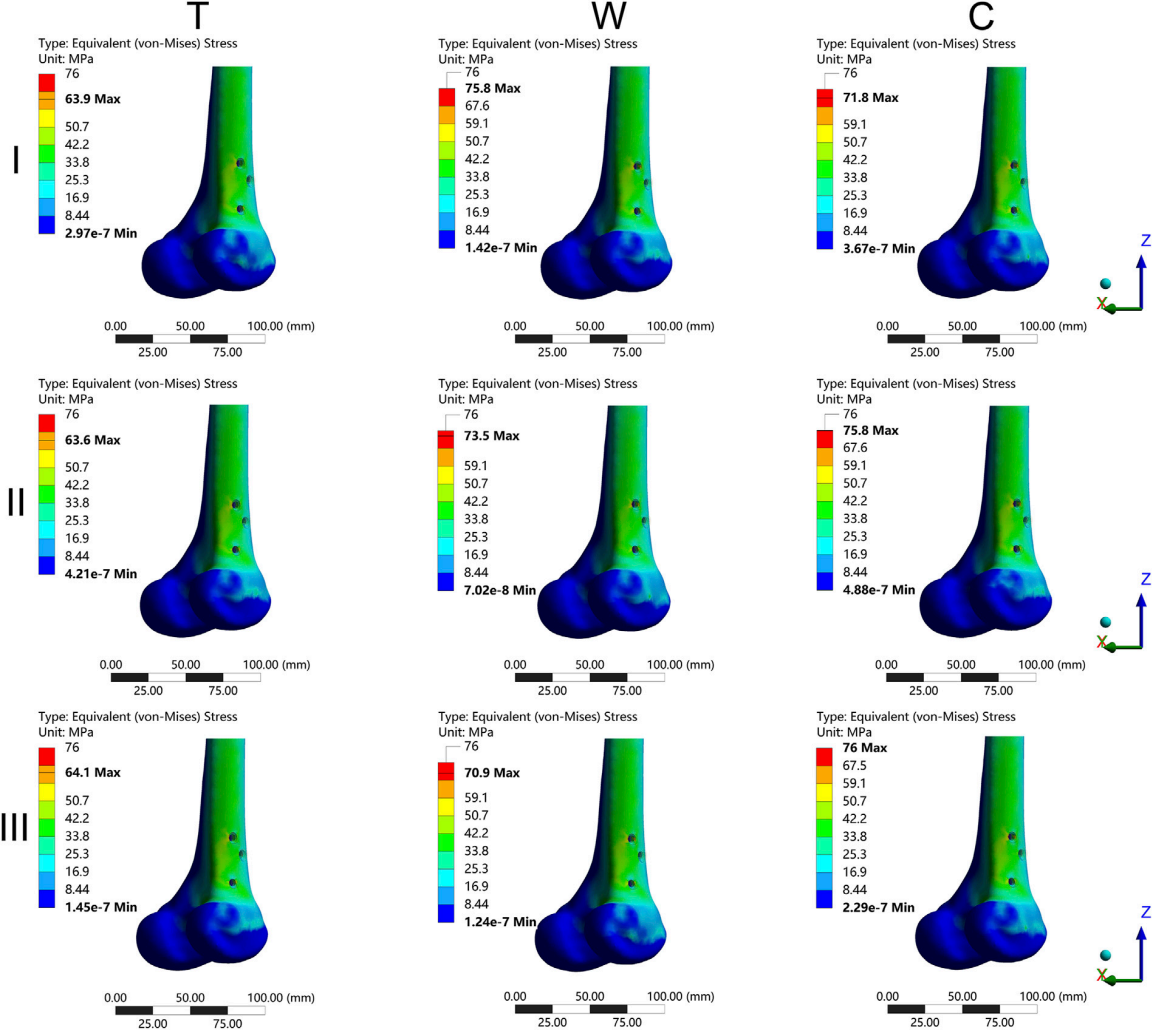
The cloud diagram of the maximum distal femur von Mises stress. I: Pauwels type I femoral neck fracture; II: Pauwels type II femoral neck fracture; III: Pauwels type III femoral neck fracture; T: Transverse femoral shaft fracture, W: wedge-shaped femoral shaft fracture, C: comminuted femoral shaft fracture.

The results of the two-way repeated measures ANOVA revealed a significant influence of both fracture type and its combination on key parameters, including MIVMS, MPFVMS, and MDFVMS (Table 1; Figures 4A–C). Specifically, MIVMS was predominantly concentrated at the fracture line of the femoral shaft under a single-leg standing load, with the III-C group exhibiting the highest value (283.4 ± 2.2 MPa) and the II-T group showing the lowest value (134.1 ± 25. MPa) (Figure 5). The reduction in MIVMS in the II-T group compared to the III-C group was 52.7%. MPFVMS was primarily concentrated below the femoral neck (Figure 6), while MDFVMS was focused at the interface between the locking screw and bone (Figure 7). The MPFVMS and MDFVMS of the III-C group were significantly high at 74.6 ± 4.8 MPa and 79.1 ± 2.7 MPa, respectively. Particularly, the MPFVMS showed a notable increase compared to the transverse and wedge FSF groups within all comminuted FSF groups, focusing specifically on the bone-nail interface of the femoral shaft fracture.

**FIGURE 8:**
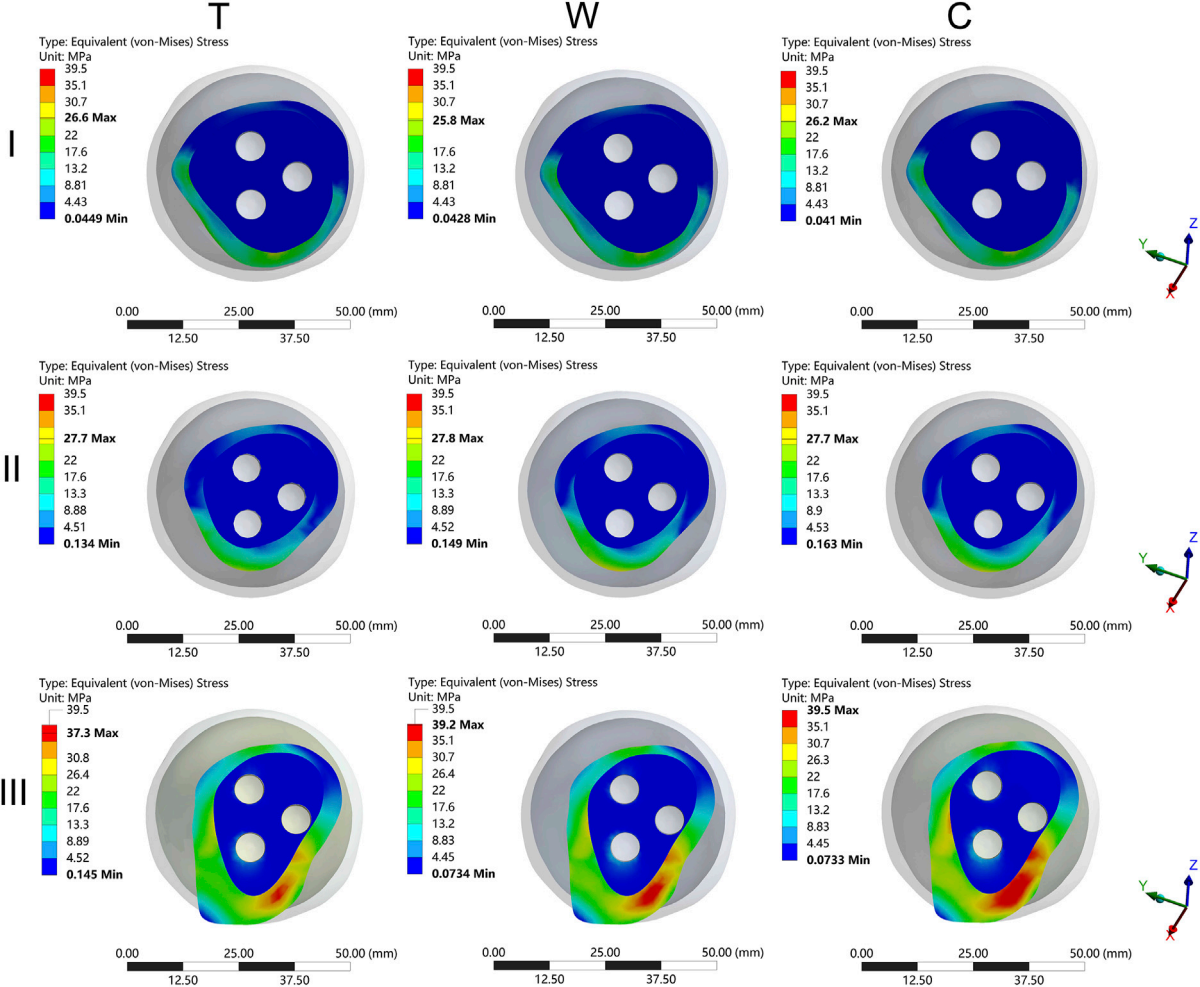
The cloud diagram of the maximum femoral neck von Mises stress. I: Pauwels type I femoral neck fracture; II: Pauwels type II femoral neck fracture; III: Pauwels type III femoral neck fracture; T: Transverse femoral shaft fracture, W: wedge-shaped femoral shaft fracture, C: comminuted femoral shaft fracture.

The MFNVMS and MFHNIM were additional parameters influenced by the type of femoral neck fracture (Table 1; Figures 4D,E, 8). The highest and lowest MFNVMS values were observed in the III-C (39.3 ± 2.4 MPa) and I-W (25.9 ± 3.6 MPa) groups, respectively, indicating a 34.1% lower stress on the neck fracture surface in the I-W group compared to the III-C group. Similarly, the highest and lowest MFHNIM values were found in the III-C (0.200 ± 0.020 mm) and I-T (0.110 ± 0.012 mm) groups, respectively, with a 45.0% reduction in MFHNIM in the I-W group compared to the III-C group.

Apart from MIVMS, MPFVMS, and MDFVMS, the MPDFIM index is also influenced by the type of FSF (Table 1; Figure 4F). The MPDFIM value was most pronounced in the III-C group at 0.271 ± 0.025 mm, whereas it was least in the I-T group at 0.011 ± 0.001 mm, indicating a 95.9% decrease relative to the III-C group.

### 3.3 Entropy scoring method

**FIGURE 9:**
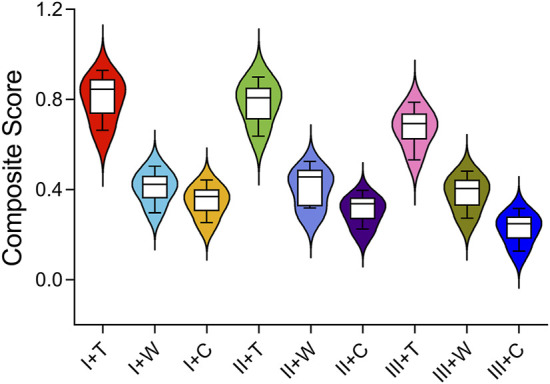
Composite scores for different types of IFN-SFs calculated using the entropy score method.

The entropy scoring method evaluated various biomechanical parameters to generate composite scores for IFN-SF models (Table 2). The MIVMS and MPDFIM were assigned the highest weights of 24.53% and 17.1%, respectively, underscoring their significance in evaluating the fixation stability of these fractures. The analysis of the composite scores revealed that the I-T group obtained the highest score of 0.79, whereas the III-C group received the lowest score of 0.22 (Figure 9).

**TABLE 2:** Entropy value, utility value, and weight of biomechanical parameters.

Parameters	Entropy value (e)	Utility value (d)	Weighting coefficient (%)
Stiffness (N/mm)	0.953	0.047	9.7
MIVMS (Mpa)	0.882	0.118	24.5
MID (mm)	0.961	0.039	8.2
MPFVMS (MPa)	0.961	0.039	8.2
MDFVMS (MPa)	0.962	0.038	7.9
MFD (mm)	0.942	0.058	12.0
MFNVMS (MPa)	0.965	0.035	7.3
MFHNIM (mm)	0.976	0.024	5.1
MPDFIM (mm)	0.918	0.082	17.1

## 4 Discussion

Despite the availability of surgical techniques and implants with varying degrees of success in treating IFN-SFs, complications related to bone healing remain prevalent. The initial stability of the fracture site significantly influences fracture healing (Burgers et al., 2011). However, existing clinical studies have predominantly concentrated on the impact of different fixation methods on initial stability, with less emphasis on the influence of fracture type (Alborno et al., 2023). This study evaluated nine biomechanical parameters related to IFN-SFs through patient-specific FEA and quantified the effect of various fracture types on fixation stability. Entropy scores derived from biomechanical parameters indicated that the MIVMS and the MPDFIM were crucial for assessing fixation stability in this fracture type. Furthermore, Type III-C fractures demonstrated the highest degree of instability, necessitating heightened attention during treatment for patients with such fractures.

The validation of the simulation model was confirmed by comparing the FEA outcomes with the biomechanical findings from a prior study. The stiffness values obtained from computer simulations for the Type II-C fracture model (321.4 ± 68.8 N/mm) were found to align with the biomechanical data reported by Wu et al. (344.8 N/mm) (Wu and Tai, 2009), within ±1 standard deviation under identical boundary conditions. This alignment confirms the adequacy of the model for subsequent analysis.

### 4.1 Fracture type determined biomechanical stability of IFN-SFs fixation constructs

The spatial characteristics of a fracture, rather than isolated damage at a single site, significantly influence the biomechanical stability of fracture fixation. Our findings demonstrate that type III-C fracture fixation constructs exhibit the lowest composite score, indicating the poorest stability compared to other fracture types. FEA results suggest that the FNF type significantly influences the overall fixation stability. In cases of type III-C fractures with a Pauwels angle exceeding 50°, the transmission of load from the femoral head through the calcar to the femoral shaft is inefficient, necessitating reliance on implant transmission. This leads to concentrated implant stress at the fracture line of the femoral shaft, aligning with the site of fixation failure observed in clinical practice (Zhang et al., 2021).

The types of FSFs work together to affect the overall stability of the fracture fixation. Transverse fractures provide a large contact area, maintaining the MPDFIM within the optimal range for bone healing. Wedge-shaped fractures maintain partial cortical contact and exhibit intermediate stability between transverse and comminuted fractures. Comminuted FSFs, however, pose a higher risk of nonunion due to elevated MPDFIM values. These observations align with the retrospective clinical analysis by Lin et al. (2014), which demonstrated a correlation between the fracture gap and bone healing. Their findings revealed that bone healing was approximately 3.6 times more likely in the low fracture gap group compared to the high fracture gap group at the 12-month postoperative mark.

The interaction of femoral neck and femoral shaft fractures increases the risk of deterioration of biomechanical stability of fixation constructs. The outcomes of two-way ANOVA showed that the types of IFN-SFs significantly affected the biomechanical parameters such as MIVMS, MPFVMS, and MDFVMS. Consequently, previous clinical studies suggesting no disparity in treatment efficacy between single and dual implants for IFN-SFs may have been influenced by confounding variables related to the type of fracture. In instances where a substantial number of I-T fractures are included in the analysis, distinctions in fixation techniques may be overshadowed by the inherent stability of the fracture itself.

### 4.2 Key biomechanical parameters for stability assessment of IFN-SFs

The interaction outcomes of IFN-SF types underscore the necessity for a comprehensive evaluation of multiple fractures. Traditional studies commonly rely on stress, displacement, and stiffness indicators to assess the general stability of IFN-SFs (Xiao et al., 2025). However, it is crucial to recognize that various biomechanical parameters hold distinct implications for assessing the biomechanical stability across different fracture sites. Notably, MFHNIM and MPDFIM play pivotal roles in appraising the fixation stability of FNF and FSF. The EEM score offers a comprehensive evaluation of all biomechanical parameters to assess the overall effectiveness of fracture fixation (Zhan et al., 2021). This study employed EEM to objectively assign weights to nine commonly utilized biomechanical parameters for assessing IFN-SFs, thereby establishing a multi-parameter evaluation framework. The results revealed that the MIVMS (weight = 24.53%) and MPDFIM (weight = 17.1%) emerged as primary predictors of IFN-SF fixation stability. Nonetheless, specific biomechanical parameters of the femoral neck, such as the MFHNIM, require independent evaluation in conjunction with the fracture type.

The spatial distribution of implant stress and the load-conduction path are critical factors that directly influence the longevity of implants (Zhang et al., 2025). In all categories of comminuted FSFs, the Maximum Implant Von Mises Stress (MIVMS) was predominantly localized in the region of implant-bone interface distal to the fracture end of the femoral shaft. Owing to inadequate bone support in this specific region, the MIVMS exhibited a significant elevation compared to wedge-shaped and transverse FSFs, consequently increasing the risk of fixation failure. This finding is consistent with previous research conclusions (Jitprapaikulsarn et al., 2025). Hence, for such fractures, the implementation of supplementary fixation strategies like combined wire fixation may enhance the stability of fracture fixation (Wang et al., 2021).

The mechanical performance of FSF fixation is assessed by the MPDFIM, which plays a crucial role in clinical practice. Studies have shown that the ideal interfragmentary motion at the fracture end is between 0.2 and 1 mm (Han et al., 2020). Moderate axial movement between fracture fragments can stimulate the activity of osteoblasts, while excessive movement can inhibit callus formation and lead to the generation of fibrous connections (Elkins et al., 2016). Conversely, comminuted FSFs compromise the circumferential support structure of the diaphyseal cortical bone, resulting in a notable increase in displacement between femoral shaft fragments, surpassing the ideal threshold for callus formation (Claes et al., 1998). Consistent with previous research, high-gap fractures exhibit increased interfragmentary motion and reduced fixation stability (Abd Aziz et al., 2024). Our study reveals that axial movement of femoral shaft fragments in wedge-shaped and comminuted FSF categories exceeds the threshold for optimal callus formation, thereby diminishing fracture fixation stability compared to transverse FSF cases.

The mechanical stability of fracture fixation is closely related to the MFHNIM, which also impacts blood supply. Pauwels type III FNFs showed the highest MFHNIM values among all fracture models. Inadequate stability due to excessive shear displacement impedes callus formation at the end of the FNF, raising the likelihood of non-union and femoral head necrosis (Augat et al., 2003).

By comprehensively evaluating nine biomechanical parameters to assess the fixation stability of femoral neck and shaft fractures using the entropy method, the potential bias of relying on a single indicator is avoided. The MFNVMS in the I-C group was comparable to that in the I-T group. However, the MPDFIM in the I-C group exhibited a significant increase and was notably lower than that in the I-T group, highlighting the distinct risk associated with comminuted FSFs. This underscores the importance of employing a multi-parameter approach to assess overall stability, as reliance on a single biomechanical parameter may lead to misinterpretations.

### 4.3 Clinical implications of fracture pattern-specific prognosis in IFN-SFs

The present study establishes a biomechanical foundation for enhancing the clinical outcomes of patients with IFN-SFs of varying fracture patterns. Through a quantitative analysis of the biomechanical stability across different fracture types, a system for assessing “fracture type-stability risk” in clinical settings has been developed. Clinical evidence reveals that in stable IFN-SFs, such as I-T type fractures, there was no significant disparity in healing rates between the single-implant and double-implant groups (Singh et al., 2008). This aligns with the current study’s results, indicating that the intrinsic stability of the fracture may mitigate differences arising from various fixation methods. Conversely, in unstable IFN-SFs, the healing rates of femoral neck and shaft fractures were notably higher in the double-implant group compared to the single-implant group (Ostrum et al., 2014). Hence, for such fractures, the selection of double implants, precise reduction achievement, and reinforcement of fixation during surgery are imperative strategies to reduce implant stress, minimize femoral head-neck interfragmentary motion, and enhance fracture fixation stability (Oh et al., 2021). Furthermore, in cases of comminuted FSFs, reducing the residual gap at the fracture site and restoring cortical continuity are critical for enhancing fixation stability. In instances where a substantial fracture gap persists following reduction, the consideration of artificial bone grafting or biodegradable three-dimensional printed support cages is warranted to enhance fracture stability and promote bone healing (Chou et al., 2017). Unstable IFN-SFs have shown to surpass the bone healing threshold under simulated single-leg weight-bearing loads, indicating the importance of avoiding such loading conditions postoperatively for these patients. For low-risk Type I-T fractures, stabilizing the fracture fixation can be achieved effectively by using a reconstruction nail in combination with cannulated screws. However, it is crucial to perform a pre-surgery hip MRI to identify any hidden FNFs (MacKinnon et al., 2022), which can be obscured by high-energy injuries such as shaft fractures. Despite the use of high-resolution CT scans in the initial evaluation of IFN-SFs, up to 30% of cases may overlook FNFs, leading to substantial medical challenges for patients (Tornetta et al., 2007). The varied biomechanical stability exhibited by different FNF types highlights the significance of promptly recognizing the fracture classification.

### 4.4 Limitation

This study offers novel insights into the biomechanical characteristics of different types of IFN-SFs, but it has certain limitations. Firstly, despite efforts to enhance the sample size to mitigate the influence of material property variations within individual samples on the experimental outcomes, the number of patients included remains restricted. Consequently, the trends identified in this study may not be universally generalizable. Additionally, the analysis is restricted to assessing biomechanical outcomes under a single-leg standing load condition, neglecting dynamic activities like walking and stair climbing that may lead to distinct stress distributions. Thus, additional research is warranted to investigate these scenarios further.

## 5 Conclusion

Our findings indicate that the type of FNF plays a crucial role in determining fixation stability in IFN-SFs, with comminuted FSFs being a significant contributing factor. The MIVMS and MPDFIM serve as essential parameters for assessing the overall stability of these fractures. Specifically, Pauwels type III FNF and comminuted FSF represent a biomechanically high-risk subtype that requires personalized and multimodal fixation approaches.

## Data Availability

The raw data supporting the conclusions of this article will be made available by the authors, without undue reservation.

## Abbreviations

IFN-SFs, ipsilateral femoral neck and shaft fractures; FEA, finite element analysis; FNFs, femoral neck fractures; FSFs, femoral shaft fractures; CT, computed tomography; I-T, Pauwels type I femoral neck fracture and transverse femoral shaft fracture; I-W, Pauwels type I femoral neck fracture and wedge-shaped femoral shaft fracture; I-C, Pauwels type I femoral neck fracture and comminuted femoral shaft fracture; II-T, Pauwels type II femoral neck fracture and transverse femoral shaft fracture; II-W, Pauwels type II femoral neck fracture and wedge-shaped femoral shaft fracture; II-C, Pauwels type II femoral neck fracture and comminuted femoral shaft fracture; III-T, Pauwels type III femoral neck fracture and transverse femoral shaft fracture; III-W, Pauwels type III femoral neck fracture and wedge-shaped femoral shaft fracture; III-C, Pauwels type III femoral neck fracture and comminuted femoral shaft fracture; HU, Hounsfield Unit; VMS, Von Mises stress; MIVMS, maximum implant von Mises stress; MID, maximum implant displacement; MPFVMS, maximum proximal femur von Mises stress; MDFVMS, maximum distal femur von Mises stress; MFD, maximum femur displacement; MFNVMS, maximum femoral neck von Mises stress; MFHNIM, maximum femoral head-neck interfragmentary motion; MPDFIM, maximum proximal-distal femur interfragmentary motion.

## References

[B1] Abd Aziz A. U. Ammarullah M. I. Ng B. W. Gan H. S. Abdul Kadir M. R. Ramlee M. H. (2024). Unilateral external fixator and its biomechanical effects in treating different types of femoral fracture: a finite element study with experimental validated model. Heliyon 10 (4), e26660. 10.1016/j.heliyon.2024.e26660 38404809 PMC10884926

[B2] Alborno Y. Abunimer A. Abuodeh Y. Salameh M. Kayali H. Ahmed G. (2023). The surgical outcomes of fixing ipsilateral femoral neck and shaft fractures: single versus double implants fixation. Eur. J. Orthop. Surg. Traumatol. 33 (5), 1613–1618. 10.1007/s00590-022-03312-0 35781618 PMC10276091

[B3] Augat P. Burger J. Schorlemmer S. Henke T. Peraus M. Claes L. (2003). Shear movement at the fracture site delays healing in a diaphyseal fracture model. J. Orthop. Res. 21 (6), 1011–1017. 10.1016/s0736-0266(03)00098-6 14554213

[B4] Bastian J. D. Ivanova S. Mabrouk A. Biberthaler P. Caba-Doussoux P. Kanakaris N. K. (2023). Surgical fixation of ipsilateral femoral neck and shaft fractures: a matter of debate? EFORT Open Rev. 8 (9), 698–707. 10.1530/eor-23-0006 37655843 PMC10548304

[B5] Boulton C. L. Pollak A. N. (2015). Special topic: ipsilateral femoral neck and shaft fractures--does evidence give us the answer? Injury 46 (3), 478–483. 10.1016/j.injury.2014.11.021 25593045

[B6] Burgers P. T. Van Riel M. P. Vogels L. M. Stam R. Patka P. Van Lieshout E. M. (2011). Rigidity of unilateral external fixators--a biomechanical study. Injury 42 (12), 1449–1454. 10.1016/j.injury.2011.05.024 21703616

[B7] Chou Y. C. Lee D. Chang T. M. Hsu Y. H. Yu Y. H. Chan E. C. (2017). Combination of a biodegradable three-dimensional (3D) - printed cage for mechanical support and nanofibrous membranes for sustainable release of antimicrobial agents for treating the femoral metaphyseal comminuted fracture. J. Mech. Behav. Biomed. Mater 72, 209–218. 10.1016/j.jmbbm.2017.05.002 28501000

[B8] Claes L. E. Heigele C. A. Neidlinger-Wilke C. Kaspar D. Seidl W. Margevicius K. J. (1998). Effects of mechanical factors on the fracture healing process. Clin. Orthop. Relat. Res. 355 (Suppl. l), S132–S147. 10.1097/00003086-199810001-00015 9917634

[B9] Elkins J. Marsh J. L. Lujan T. Peindl R. Kellam J. Anderson D. D. (2016). Motion predicts clinical callus formation: construct-specific finite element analysis of Supracondylar femoral fractures. J. Bone Jt. Surg. Am. 98 (4), 276–284. 10.2106/jbjs.o.00684 PMC5141368 26888675

[B10] Gupta A. Jain A. Mittal S. Chowdhury B. Trikha V. (2023). Ipsilateral femoral neck and shaft fractures: case series from a single Level-I trauma centre and review of literature. Eur. J. Orthop. Surg. Traumatol. 33 (4), 803–809. 10.1007/s00590-021-03199-3 35119486

[B11] Hak D. J. Mauffrey C. Hake M. Hammerberg E. M. Stahel P. F. (2015). Ipsilateral femoral neck and shaft fractures: current diagnostic and treatment strategies. Orthopedics 38 (4), 247–251. 10.3928/01477447-20150402-05 25879185

[B12] Han Z. Wu J. Deng G. Bi C. Wang J. Wang Q. (2020). Axial Micromotion locking plate construct can promote Faster and Stronger bone healing in an ovine osteotomy model. Front. Bioeng. Biotechnol. 8, 593448. 10.3389/fbioe.2020.593448 33520953 PMC7845656

[B13] Huang X. Zhang F. Zhang Y. (2021). Case series and finite element analysis of PFNA combined with cerclage wire for treatment of subtrochanteric fracture of femur. J. Orthop. Surg. Res. 16 (1), 70. 10.1186/s13018-020-02187-3 33472679 PMC7816411

[B14] Huang Q. Zhang C. Bai H. Wang Q. Li Z. Lu Y. (2023). Biomechanical evaluation of two modified intramedullary fixation system for treating unstable femoral neck fractures: a finite element analysis. Front. Bioeng. Biotechnol. 11, 1116976. 10.3389/fbioe.2023.1116976 36896014 PMC9989215

[B15] Jian-Qiao Peng M. Chen H. Y. Ju X. Hu Y. Ayoub A. Khambay B. (2020). Comparative analysis for five fixations of Pauwels-I by the biomechanical finite-element method. J. Invest. Surg. 33 (5), 428–437. 10.1080/08941939.2018.1533054 30516078

[B16] Jitprapaikulsarn S. Chantarapanich N. Apivatthakakul T. Lertvilai P. Wanchat S. Gromprasit A. (2025). How wide of a distal metaphyseal femoral fracture gap is a high risk of varus collapse and fixation failure? A finite element study. Injury 56 (2), 112091. 10.1016/j.injury.2024.112091 39787783

[B17] Jung C. H. Cha Y. Yoon H. S. Park C. H. Yoo J. I. Kim J. T. (2022). Mechanical effects of surgical variations in the femoral neck system on Pauwels type III femoral neck fracture: a finite element analysis. Bone Jt. Res. 11 (2), 102–111. 10.1302/2046-3758.112.bjr-2021-0282.r1 PMC8882323 35168366

[B18] Lin S. J. Chen C. L. Peng K. T. Hsu W. H. (2014). Effect of fragmentary displacement and morphology in the treatment of comminuted femoral shaft fractures with an intramedullary nail. Injury 45 (4), 752–756. 10.1016/j.injury.2013.10.015 24268188

[B19] Mackinnon T. Selmi H. Davies A. Packer T. W. Reilly P. Sarraf K. M. (2022). Protocolised MRI as an adjunct to CT in the diagnosis of femoral neck fracture in high energy ipsilateral femoral shaft fractures - a break-even analysis. Injury 53 (12), 4099–4103. 10.1016/j.injury.2022.10.005 36272845

[B20] Marins M. H. T. Pallone L. V. Vaz B. a. S. Ferreira A. M. Nogueira-Barbosa M. H. Salim R. (2021). Ipsilateral femoral neck and shaft fractures. When do we need further image screening of the hip? Injury 52 (Suppl. 3), S65–s69. 10.1016/j.injury.2021.01.040 34083022

[B21] Mimata H. Matsuura Y. Yano S. Ohtori S. Todo M. (2022). Evaluation of bone healing process after intramedullary nailing for femoral shaft fracture by quantitative computed tomography-based finite element analysis. Clin. Biomech. (Bristol) 100, 105790. 10.1016/j.clinbiomech.2022.105790 36327546

[B22] Mohan K. Ellanti P. French H. Hogan N. Mccarthy T. (2019). Single versus separate implant fixation for concomitant ipsilateral femoral neck and shaft fractures: a systematic review. Orthop. Rev. (Pavia) 11 (2), 7963. 10.4081/or.2019.7963 31316738 PMC6603431

[B23] Oh C. W. Kim J. W. Oh J. K. Apivatthakakul T. Park K. H. Hong W. (2021). “Reverse miss-a-nail technique” of reconstruction nailing for successful fixation of the ipsilateral femoral neck and shaft fracture. Arch. Orthop. Trauma Surg. 141 (6), 959–969. 10.1007/s00402-020-03620-2 33040209

[B24] Ostrum R. F. Tornetta P. 3rd Watson J. T. Christiano A. Vafek E. (2014). Ipsilateral proximal femur and shaft fractures treated with hip screws and a reamed retrograde intramedullary nail. Clin. Orthop. Relat. Res. 472 (9), 2751–2758. 10.1007/s11999-013-3271-5 24014269 PMC4117883

[B25] Peng M. J. Xu H. Chen H. Y. Lin Z. Li X. Shen C. (2020). Biomechanical analysis for five fixation techniques of Pauwels-III fracture by finite element modeling. Comput. Methods Programs Biomed. 193, 105491. 10.1016/j.cmpb.2020.105491 32388067

[B26] Samsami S. Augat P. Rouhi G. (2019). Stability of femoral neck fracture fixation: a finite element analysis. Proc. Inst. Mech. Eng. H. 233 (9), 892–900. 10.1177/0954411919856138 31203740

[B27] Singh R. Rohilla R. Magu N. K. Siwach R. Kadian V. Sangwan S. S. (2008). Ipsilateral femoral neck and shaft fractures: a retrospective analysis of two treatment methods. J. Orthop. Traumatol. 9 (3), 141–147. 10.1007/s10195-008-0025-3 19384610 PMC2656981

[B28] Spitler C. A. Kiner D. Swafford R. Bruce J. Nowotarski P. (2020). Treatment of ipsilateral femoral neck and shaft fractures with cannulated screws and Antegrade reconstruction nail. J. Orthop. Trauma 34 (5), e176–e180. 10.1097/bot.0000000000001689 31688439

[B29] Tornetta P. 3rd Kain M. S. Creevy W. R. (2007). Diagnosis of femoral neck fractures in patients with a femoral shaft fracture. Improvement with a standard protocol. J. Bone Jt. Surg. Am. 89 (1), 39–43. 10.2106/00004623-200701000-00006 17200308

[B30] Wang T. H. Chuang H. C. Kuan F. C. Hong C. K. Yeh M. L. Su W. R. (2021). Role of open cerclage wiring in patients with comminuted fractures of the femoral shaft treated with intramedullary nails. J. Orthop. Surg. Res. 16 (1), 480. 10.1186/s13018-021-02633-w 34364374 PMC8348994

[B31] Wu C. C. Tai C. L. (2009). Reconstruction interlocking nails for ipsilateral femoral neck and shaft fractures: biomechanical analysis of effect of supplementary cannulated screw on intracapsular femoral neck fracture. Clin. Biomech. (Bristol) 24 (8), 642–647. 10.1016/j.clinbiomech.2009.06.009 19635640

[B32] Xia Y. Zhang W. Hu H. Yan L. Zhan S. Wang J. (2021). Biomechanical study of two alternative methods for the treatment of vertical femoral neck fractures - a finite element analysis. Comput. Methods Programs Biomed. 211, 106409. 10.1016/j.cmpb.2021.106409 34560605

[B33] Xiao Y. He K. Tan X. Wei D. Yan J. Yang Y. (2025). Biomechanical evaluation of implant techniques for ipsilateral femoral neck and shaft fractures: a finite element analysis. J. Mech. Behav. Biomed. Mater 164, 106890. 10.1016/j.jmbbm.2025.106890 39971414

[B34] Yuan D. Wu Z. Luo S. Zhou Y. Teng J. Ye C. (2022). Improve biomechanical stability using intramedullary nails with femoral neck protection in femoral shaft fractures. Comput. Methods Programs Biomed. 225, 107078. 10.1016/j.cmpb.2022.107078 36037604

[B35] Zeng W. Liu Y. Hou X. (2020). Biomechanical evaluation of internal fixation implants for femoral neck fractures: a comparative finite element analysis. Comput. Methods Programs Biomed. 196, 105714. 10.1016/j.cmpb.2020.105714 32858283

[B36] Zhan S. Jiang D. Ling M. Ding J. Yang K. Duan L. (2021). Fixation effects of different types of cannulated screws on vertical femoral neck fracture: a finite element analysis and experimental study. Med. Eng. Phys. 97, 32–39. 10.1016/j.medengphy.2021.09.007 34756336

[B37] Zhang W. Antony Xavier R. P. Decruz J. Chen Y. D. Park D. H. (2021). Risk factors for mechanical failure of intertrochanteric fractures after fixation with proximal femoral nail antirotation (PFNA II): a study in a Southeast Asian population. Arch. Orthop. Trauma Surg. 141 (4), 569–575. 10.1007/s00402-020-03399-2 32296964

[B38] Zhang X. Zhang S. Zhong Z. Zhang W. Xiong Z. (2025). Computational evaluation of the biomechanical effects of position changes in the femoral neck system on Pauwels type III femoral neck fractures: an *in silico* study. Front. Bioeng. Biotechnol. 13, 1493555. 10.3389/fbioe.2025.1493555 40066362 PMC11891373

[B39] Zhao Y. Li J. Liu Y. Cui G. Li Z. (2023). Comparison of reconstruction nails versus dual implants in the treatment of ipsilateral femoral neck and shaft fractures in adults: a meta-analysis and systematic review. BMC Musculoskelet. Disord. 24 (1), 800. 10.1186/s12891-023-06933-6 37814281 PMC10561477

[B40] Zhong Z. Lan X. Xiang Z. Duan X. (2023). Femoral neck system and cannulated compression screws in the treatment of non-anatomical reduction Pauwels type-III femoral neck fractures: a finite element analysis. Clin. Biomech. (Bristol) 108, 106060. 10.1016/j.clinbiomech.2023.106060 37536196

