# Supplementary material for: Quantitative biomechanical analysis of fracture patterns in ipsilateral femoral neck and shaft fractures: an in-silico study
Source: Front Bioeng Biotechnol. 2025 Aug 22;13:1641700. doi: 10.3389/fbioe.2025.1641700 (PMC12411502; doi:10.3389/fbioe.2025.1641700)
Supplement: Supplementary file 1 [file DataSheet1.docx]

**Supplementary Tables**

**Supplementary Table 1.** Baseline information for participants.

| Participants | Age | Gender | BMI (Kg/cm2) | E _Cortical bone_ (MPa) | E _Cancellous bone_ (MPa) | Neck-Shaft Angle |
| --- | --- | --- | --- | --- | --- | --- |
| 1 | 39 | M | 24.1 | 11355.3 | 668.4 | 123.6 |
| 2 | 42 | M | 19.4 | 18641.8 | 1076.3 | 132.5 |
| 3 | 44 | M | 14.7 | 13655.3 | 900.1 | 122.4 |
| 4 | 27 | M | 23.9 | 11191.0 | 560.9 | 127.8 |
| 5 | 33 | M | 22.5 | 15252.7 | 801.3 | 131.6 |
| 6 | 36 | M | 19.5 | 16565.7 | 668.4 | 137.3 |
| 7 | 34 | F | 23.7 | 17873.6 | 1015.4 | 126.8 |
| 8 | 54 | F | 21.5 | 16616.7 | 835.4 | 121.7 |
| 9 | 48 | F | 22.9 | 12995.0 | 773.6 | 122.8 |
| 10 | 37 | F | 22.5 | 17527.0 | 653.4 | 125.9 |

**Supplementary Table 2.** Mesh convergence analysis of the maximum von Mises stress in relation to mesh size for implant and bone models. Taking ipsilateral Pauwels type II femoral neck fracture and comminuted femoral shaft fracture as an example.

| **Mesh size (mm)** | **1** | **2** | **3** | **4** | **5** |
| --- | --- | --- | --- | --- | --- |
| MIVMS*^a^* (MPa) | 285.3 | 282.2 | 279.6 | 260.5 | 240.8 |
| MFVMS *^b^*(MPa) | 69.2 | 70.8 | 68.7 | 63.2 | 58.6 |
| Computing time (s) | 2825 | 785 | 492 | 374 | 128 |

*^a^* MIVMS: maximum implant von Mises stress.*^b^* MFVMS: maximum femur von Mises stress.

**Supplementary Figure**

Supplementary Figure 1. Convergence analysis of mesh size.

**
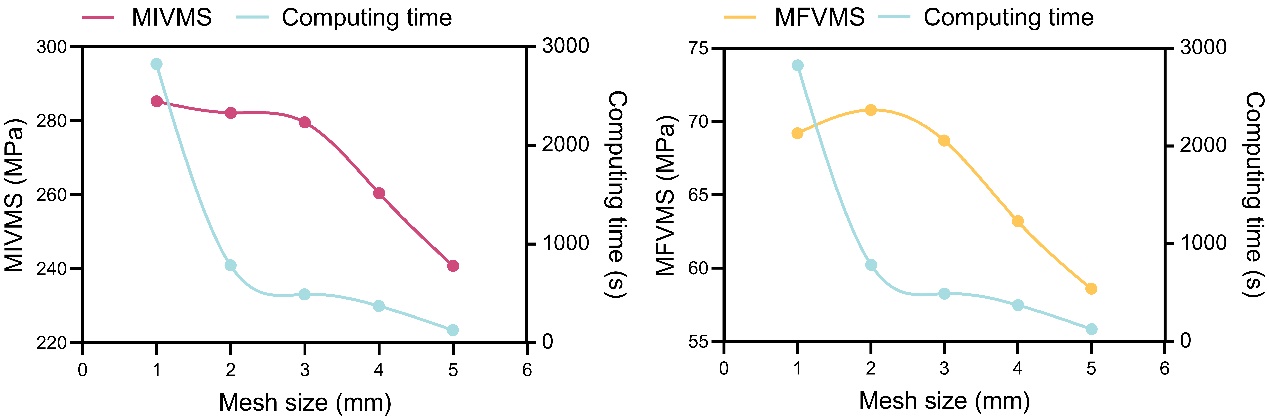
**
